# Late presentation of hyperandrogenism in pregnancy: clinical features and differential diagnosis

**DOI:** 10.1530/EDM-13-0048

**Published:** 2013-10-16

**Authors:** Gautam Das, Vinay S Eligar, Jyothish Govindan, D Aled Rees

**Affiliations:** University Hospital of WalesCardiff, CF14 4XWUK

## Abstract

**Background:**

Hyperandrogenic states in pregnancy are rare but arise most commonly due to new-onset ovarian pathology in pregnancy. We describe the case of a young woman who presented in the latter half of her pregnancy with features of hyperandrogenism. We review the biochemical and imaging findings and discuss the differential diagnosis.

**Case presentation:**

A 26-year-old woman presented in the later part of her pregnancy with widespread hirsutism. Biochemical testing confirmed hyperandrogenism (testosterone, 13.7 nmol/l and second-trimester pregnancy range, 0.9–4.9 nmol/l), although she had no history of menstrual disturbance, hirsutism or acne prior to conception. Radiological evaluation (ultrasound and magnetic resonance imaging) revealed multiple cystic lesions in both ovaries, leading to a presumptive diagnosis of hyperreactio luteinalis (HL). The implications of maternal hyperandrogenism on foetal virilisation were considered and the patient was counselled appropriately. She delivered a healthy baby boy uneventfully. Androgen levels, hirsutism and acne normalised within a few weeks of delivery.

**Conclusion:**

HL can occur at any stage of pregnancy and is an important differential diagnosis in pregnant patients with features of androgen excess. Most cases regress spontaneously after delivery and major interventions are usually not needed.

**Learning points:**

- Hyperandrogenism in pregnancy is rare.
- Clinical features are similar to the non-pregnant state in the mother but virilisation in the foetus can have profound consequences.
- HL and pregnancy luteoma are the most common ovarian pathologies leading to hyperandrogenism in pregnancy.
- Spontaneous regression occurs in the *post-partum* period in the vast majority of cases and surgery is only required for local complications.

## Background

Hyperandrogenism presenting during pregnancy usually arises due to pregnancy-related conditions, as pre-existing high androgen levels typically lead to anovulation and infertility (1). These conditions are rare and can occur at any stage of pregnancy. The outcome can range from an uneventful pregnancy to varying degrees of foetal or maternal virilisation depending on the underlying aetiology. Hyperreactio luteinalis (HL) and pregnancy luteoma (PL) are the most frequent ovarian causes of hyperandrogenism in pregnancy, in contrast to foetal aromatase deficiency, ovarian tumours and adrenal pathologies, which are extremely rare.

## Case presentation

A 26-year-old primigravida presented at 27 weeks of gestation with symptoms of facial and abdominal hirsutism, deepening of the voice and facial acne. She had a history of normal menstrual cycles since menarche and had no other systemic complaints. She had no significant medical or drug history and the family history did not reveal any complications relating to pregnancy in her mother or sister. She was non-obese, normotensive and did not appear cushingoid but had evidence of hirsutism on her face and abdomen, acneiform lesions on her cheeks and acanthosis nigricans around the axillary regions.

## Investigation

**Figure 1:**
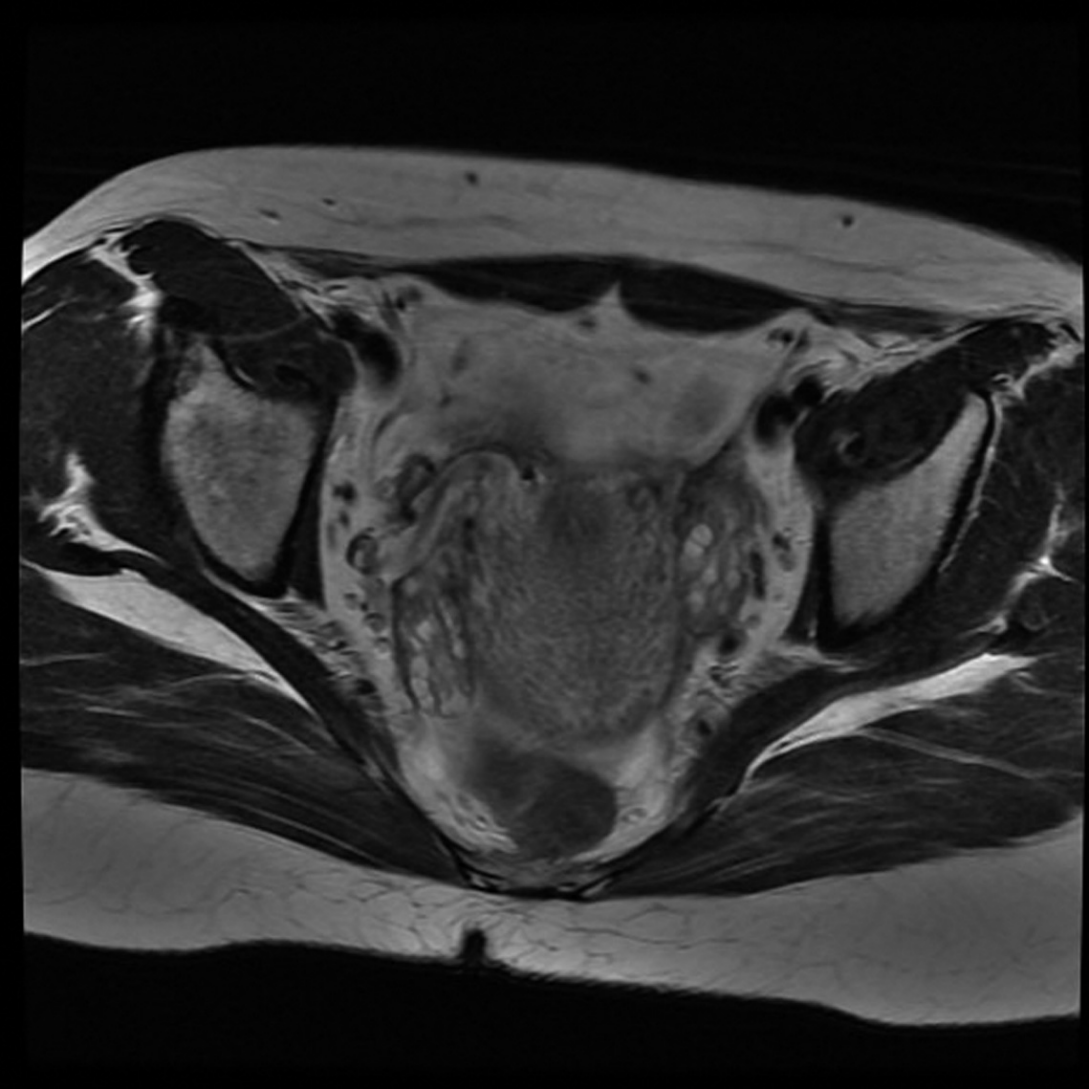
MRI scan showing gravid uterus and bilateral cystic ovaries.

Laboratory findings revealed a normal full blood count, renal function, electrolytes, liver function, thyroid function and glucose tolerance (0 min, 4.3 mmol/l and 120 min, 5.7 mmol/l). Her 17-hydroxyprogesterone (17-OHP) levels were elevated at 54.2 nmol/l (non-pregnancy range: follicular, 1–10 nmol/l and luteal, 1–20 nmol/l) in accordance with pregnancy. Serum DHEAS was normal (5.4 μmol/l; non-pregnancy range, 1.9–9.4 μmol/l) but both androstenedione (>35.3 nmol/l; non-pregnancy range, 3.0–9.6 nmol/l) and testosterone (13.7 nmol/l; second trimester pregnancy range, 0.9–4.9 nmol/l) were significantly elevated. Repeat testing a month later revealed a further increase in testosterone levels (20 nmol/l). Abdominal ultrasound showed bulky ovaries and multiple peripheral cysts, not considered typical of polycystic ovaries, and a single live male foetus. A magnetic resonance imaging (MRI; Fig. 1) scan showed similar findings and normal appearances to the adrenal glands. On the basis of these findings, a presumptive diagnosis of HL was made.

## Outcome and follow-up

The subject delivered a healthy male baby at 39 weeks of gestation. A karyotype was not undertaken as the foetus was phenotypically male with normally sited testes. She could not breastfeed for the first month and developed an irregular menstrual cycle. Assessment at that point showed evidence of ongoing hirsutism but her androgen profile improved (DHEAS, 3.2 μmol/l and androstenedione, 10 nmol/l), including normalisation of testosterone (1.7 nmol/l). Subsequently, she resumed a normal menstrual cycle with regression in hirsutism and acne. A further assessment 9 months after delivery showed complete normalisation of her androgens (DHEAS, 2.1 μmol/l and testosterone, 0.8 nmol/l) and 17-OHP levels (1.2 nmol/l).

## Discussion

Hyperandrogenism in pregnancy is rare and can develop in any trimester. The clinical symptoms for the mother may not be significantly different from that of the non-pregnant state but the foetus may be born with or without virilising features depending on the cause of the androgen excess.

Pregnancy induces several physiological changes in the mother. In normal pregnancy, an increase in maternal testosterone occurs during the first (reference range, 0.6–4.9 nmol/l) and second (reference range, 0.9–4.9 nmol/l) trimesters (2). The rise in testosterone triggers protective mechanisms to reduce excess androgen exposure in the mother and the foetus. First, an elevated level of maternal oestrogen promotes an increased synthesis of sex hormone binding globulin, which binds to testosterone and dihydrotestosterone (3), thus reducing free androgen exposure. Secondly, placental aromatase (cytochrome P450) plays a dominant role in nullifying the effects of excess DHEAS obtained from the maternal and foetal adrenal glands. DHEAS is desulphated by steroid sulphatase and subsequently converted by 3β-hydroxysteroid dehydrogenase (HSD) and 17β-HSD to androstenedione and testosterone respectively. Placental aromatase converts these androgens into oestradiol, which is eventually converted into oestriol by the foetal liver and excreted in the maternal urine (1). Elevated androgens induce increased production of placental aromatase but this system can be overcome if androgen levels are particularly high. Such a scenario, with consequent risk of virilisation of a female foetus (4), is more likely in severe hyperandrogenism as occurs with ovarian tumours (5).

The most common ovarian pathologies that present during pregnancy and which lead to hyperandrogenic states are HL and PL. HL is a relatively rare condition causing marked bilateral cystic enlargement of the ovaries, although unilateral disease is also reported (6). On ultrasound, HL appears as large adnexal masses with a ‘spoke wheel’ appearance. HL is thought to arise as a consequence of ovarian hyperstimulation from β-hCG leading to hypertrophy and luteinisation of the theca interna cells (1). The risk of HL may thus be increased in gestational trophoblastic disease, multiple pregnancies and chronic kidney diseases, the latter due to reduced clearance of β-hCG (7) (8). Most patients are asymptomatic and are detected incidentally in the third trimester by ultrasound or even after birth following delivery by caesarean section (9). HL usually manifests in primigravidae, as in our case, and most cases are reported in Caucasians. Hyperandrogenism can occur in 20–30% of cases, leading to maternal virilisation. However, foetal virilisation has never been reported (10), presumably as the extent of hyperandrogenism is insufficient to outstrip placental aromatase activity. Secondary complications such as an acute abdomen due to ovarian torsion and haemorrhage can occur (11), and reports have also linked it to ascites and pleural effusion (12). Patients can usually be managed conservatively as ovarian volume and androgen concentrations generally return to normal within 3 months of delivery. Recurrence in subsequent pregnancy is rare (13). The commonest clinical condition that may mimic HL is ovarian hyperstimulation syndrome (OHSS), which usually manifests in the first trimester. It is usually associated with ovulation induction and excess gonadotrophins but can rarely occur in a spontaneous ovulatory cycle with singleton pregnancy, multiple pregnancies, molar pregnancy, hypothyroidism and in polycystic ovary syndrome (PCOS) (14). Virilisation is exceptionally rare in OHSS but dramatic fluid shift prompting hypovolaemia and thrombosis can occur (15).

PL can also cause hyperandrogenism in pregnancy, but in contrast to HL, it is most commonly reported in Afro-Caribbeans, in women over 30 years and in those with pre-existing PCOS (16). PLs are benign non-tumourous lesions of the ovaries that arise due to the proliferation of luteinised cells under the influence of β-hCG (17). However, although β-hCG is considered aetiologically relevant, luteomas are rarely found in association with trophoblastic disease, hence other factors are likely to be important in development (18). Macroscopically, PLs are multi-nodular brown–yellow masses, 6–20 cm in size, with haemorrhagic spots (19). Microscopically, they are formed of groups of luteinised cells with large eosinophilic cytoplasm and regular nuclei (4). As with HL, the majority of PLs are discovered incidentally during pregnancy on ultrasound and are asymptomatic (20). They present with a mass in 90% of cases and should be included in the differential diagnosis of pelvic mass in pregnancy. Hypersecretion of androgens occurs in ∼25% of women, of whom 10–50% show clinical signs of hyperandrogenism. Between 60 and 70% of female infants born to masculinised mothers will have some degree of virilisation (21) (22). PL can also be associated with acute abdomen due to ovarian torsion, tumour rupture or bleeding (17), in addition to compression of surrounding structures. *Post-partum*, PLs usually regress spontaneously with normalisation of androgen levels within 2 weeks and clinical signs within 3 months (23). The suppressive effects of excess androgens on lactation may transiently impair breastfeeding (24) but PL is generally seen as a self-limiting disease, hence a conservative approach with observation alone is usually preferred. If surgery is necessary due to secondary complications, then this is best performed during the second trimester. PL has been linked to miscarriages during the first trimester and premature birth during the third trimester. Furthermore, a high recurrence rate is expected during subsequent pregnancies with further hyperandrogenism (4). Pre-implantation selection of a male embryo has been employed in such circumstances to prevent virilisation of a female foetus (25).

Other causes of hyperandrogenism during pregnancy include worsening of symptoms in patients with pre-existing PCOS, which usually manifests during the first trimester, benign and malignant tumours of the ovaries, which invariably cause virilisation, maternal exposure to androgenic drugs, congenital adrenal hyperplasia, maternal or foetal adrenal tumours and placental aromatase deficiency.

Although we consider that HL is the likeliest cause in our patient as she was a young primigravida who presented with features of hyperandrogenism in the latter part of pregnancy, had demonstrable cystic lesions in her ovaries and normalised her androgen levels within a few months after delivery, some atypical features were apparent, including less profound ultrasonic appearances than those reported in the literature and her Asian ethnicity. However, no history of exposure to androgenic drugs was elicited, *post-partum* 17-OHP levels were normal (excluding congenital adrenal hyperplasia), and imaging of the adrenal glands and ovaries did not show signs of neoplasia.

In conclusion, elevated levels of androgens in a pregnant female are rare and usually reflect a new-onset ovarian pathology as a consequence of pregnancy. HL and PL are the commonest pathologies encountered but other more serious diseases may occasionally present. A careful evaluation is required to avoid unnecessary surgery as the majority of cases resolve spontaneously. Clinicians should be reminded of the consequences that elevated androgens can have on a pregnant mother and her child and of the importance in dealing with these issues with utmost sensitivity.

## Patient consent

Written informed consent has been obtained from the patient for publication of this (anonymised) submitted case report and accompanying images.

## Author contribution statement

The article was conceived and written by G Das with expert supervision from the consultant in charge (D A Rees) of the patient at each step. J Govindan and V Eligar had substantial roles to play in organising the relevant investigations for the patient and monitoring her on an outpatient basis. They also contributed in framing up the final draft of the article. The authors have permission from D A Rees, who was responsible for this patient's care, for publishing this article.
